# Ecological convergence in phytochemistry and flower–insect visitor interactions along an Andean elevation gradient

**DOI:** 10.1002/ece3.10418

**Published:** 2023-08-16

**Authors:** Alma Nalleli Carvajal Acosta, Ludovico Formenti, Adrienne Godschalx, Angelos Katsanis, Constanza Schapheer, Kailen Mooney, Cristian Villagra, Sergio Rasmann

**Affiliations:** ^1^ Department of Entomology Michigan State University East Lansing Michigan USA; ^2^ Department of Ecology & Evolutionary Biology University of California, Irvine Irvine California USA; ^3^ Institut für Ökologie und Evolution Universität Bern Bern Switzerland; ^4^ Institute of Biology University of Neuchâtel Neuchâtel Switzerland; ^5^ Soil Food Web School Corvallis Oregon USA; ^6^ Instituto de Entomología Universidad Metropolitana de Ciencias de la Educación Santiago Chile

**Keywords:** Andean biodiversity, ecological gradients, macroecology, specialized metabolism, tephritid flies, terpenes, volatile organic compounds

## Abstract

The diversity of specialized molecules produced by plants radiating along ecological gradients is thought to arise from plants' adaptations to local conditions. Therefore, closely related species growing in similar habitats should phylogenetically converge, or diverge, in response to similar climates, or similar interacting animal communities. We here asked whether closely related species in the genus *Haplopappus* (Asteraceae) growing within the same elevation bands in the Andes, converged to produce similar floral odors. To do so, we combine untargeted analysis of floral volatile organic compounds with insect olfactory bioassay in congeneric *Haplopappus* (Asteraceae) species growing within the same elevation bands along the Andean elevational gradient. We then asked whether the outcome of biotic interactions (i.e., pollination vs. seed predation) would also converge across species within the same elevation. We found that flower odors grouped according to their elevational band and that the main floral visitor preferred floral heads from low‐elevation band species. Furthermore, the cost–benefit ratio of predated versus fertilized seeds was consistent within elevation bands, but increased with elevation, from 6:1 at low to 8:1 at high elevations. In the light of our findings, we propose that climate and insect community changes along elevation molded a common floral odor blend, best adapted for the local conditions. Moreover, we suggest that at low elevation where floral resources are abundant, the per capita cost of attracting seed predators is diluted, while at high elevation, sparse plants incur a higher herbivory cost per capita. Together, our results suggest that phytochemical convergence may be an important factor driving plant–insect interactions and their ecological outcomes along ecological gradients.

## Graphical abstract

In this study, we detected patterns of ecological convergence along elevation in floral volatiles as well as cost–benefit ratio in seed versus pollinated seeds. Our results suggest that these evolutionary patterns are the result of biotic and abiotic pressures.

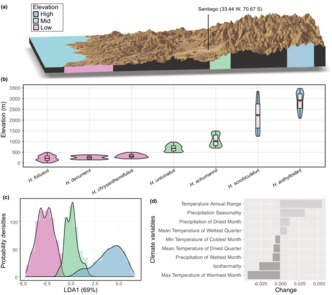

## Resumen

La gran diversidad de moléculas especializadas producidas por plantas a lo largo de gradientes ecológicos se atribuye a la adaptación de plantas a sus condiciones locales. Por tanto, especies de plantas estrechamente relacionadas que crecen en hábitats similares deberían de converger en la producción de fitoquímicos similares, en respuesta a climas similares o comunidades de animales con las que interactúan. En este estudio exploramos esta hipótesis caracterizando la metabolómica no dirigida de compuestos orgánicos volátiles florales y conduciendo bioensayos olfativos de insectos en especies cogenéticas del género *Haplopappus* (Asteraceae) que crecen dentro de las mismas bandas altitudinales a lo largo de un gradiente altitudinal andino. Conjuntamente investigamos si el resultado de las interacciones bióticas (ej. polinización versus depredación) también convergen entre especies que crecen dentro de la misma banda altitudinal. Encontramos que los olores de las flores se agrupan de acuerdo con su banda altitudinal, y que el visitante floral más común prefiere los capítulos florales de las especies de bandas de baja elevación. Además, la relación entre el costo (depredación) y beneficio (polinización) es consistente dentro de las bandas de elevación pero incrementa con elevación, de 6:1 en elevaciones bajas a 8:1 en elevaciones altas. Por lo tanto, proponemos que los cambios climáticos y la comunidad de insectos a lo largo de la elevación resultaron en una mezcla común de olores de flores, mejor adaptada a las condiciones locales. Asimismo, sugerimos que a baja altura donde los recursos florales son abundantes, el costo per cápita de atraer a los depredadores de semillas se diluye, mientras que en sitios altos, las plantas escasas incurren en un costo per cápita de herbivoría más alto. Nuestros resultados sugieren que la convergencia fitoquímica puede ser un factor importante que impulsa las interacciones planta‐insecto y sus resultados ecológicos a lo largo de gradientes ecológicos.

## 1 INTRODUCTION

Volatile organic compounds (VOCs) emitted by flowers are key signals for generating and maintaining plant interactions with animals, which can range from costly plant–herbivore and plant–seed predator interactions to beneficial plant–pollinator interactions, among others (Bakhtiari et al., 2021; Zhou & Jander, 2022). Floral VOCs, particularly those related to pollinator attraction, are highly diverse across species and habitats (Knudsen & Gershenzon, 2006). Yet, to date, ecologists and evolutionary biologists still struggle to disentangle not only the origin of VOC diversity across taxa, but also to quantify the ecological consequences of VOC diversity across species and ecosystems (Wetzel & Whitehead, 2020). It has been proposed that the production of different VOCs is the result of the plants' evolutionary history as well as the local environment (Farré‐Armengol et al., 2020). Specifically, two non‐mutually exclusive hypotheses can be proposed regarding the identity and abundance of VOCs produced by plants. On the one hand, the production of floral odor bouquets is phylogenetically constrained in which during speciation, the biosynthetic pathways for VOCs production remain stable (Knudsen & Gershenzon, 2006; Steiner et al., 2011). Under this framework, closely related lineages should display more similar floral VOCs than distantly related species, regardless of their current abiotic environment. Alternatively, VOC biosynthesis may be labile and evolve rapidly, in which case the fragrances produced by flowers reflect local adaptation to abiotic and biotic conditions leading to convergence among different species occupying the same environment (Friberg et al., 2019).

The hypotheses of phylogenetic constraint versus ecological convergence represent two theoretical extremes; the reality is likely an intermediate dynamic in which both processes shape floral fragrance (Raguso, 2008; Schwery et al., 2023). While some comparative studies have found phylogenetic structuring of floral scents (Azam et al., 2013; Steiner et al., 2011), others have found floral scent divergence driven by local biogeographic differences (Moré et al., 2021). For instance, both biotic factors, such as the relative abundance of pollinators, herbivores, or microbial pathogens (Theis et al., 2007), as well as abiotic factors (i.e., climatic conditions, physical habitat structure; Tohge et al., 2016) have been implicated in the evolution of floral scents and their functions regarding ecological interactions, with attractant (Chess et al., 2008) or defensive properties (Paul et al., 2021; reviewed in Farré‐Armengol et al., 2020). For instance, in the fig‐fig wasp association, evolutionary divergence in VOC emissions has been shown to be a key mechanism for maintaining host‐specificity along elevational gradients (Souto‐Vilarós et al., 2018) and across geographic scales (Soler et al., 2011). Therefore, one might argue that the odor blend produced by flowers of a given species should be the optimal compromise given the local biotic (i.e., for attracting pollinators while fending off seed predators; Nunes et al., 2016; Theis et al., 2007), and abiotic conditions (i.e., producing VOCs that meet optimal volatility given the surrounding average temperature and precipitation regimes; Farré‐Armengol et al., 2014; Stiles et al., 2007).

As flowering plants interact with multiple insect visitors whose function may vary from mutualists to antagonists, the fitness outcome of these biotic interactions can be expressed as a cost–benefit ratio defined as the ratio between the number of viable seeds obtained through pollination versus the number of seeds lost to herbivory (seed predation). To address the context dependency of such biotic interactions, including plant–herbivore and pollinator interactions, ecologists capitalize on the predictable environmental variation that naturally exists along large‐scale ecological gradients (Körner, 2007; Schemeske et al., 2009). For example, it is predicted that milder environmental conditions promote stronger biotic interactions and intensify seed predation (Rasmann et al., 2018). These predictions have been corroborated, for a plant–seed predator system within the climatic gradient spanning the coastal to inland distribution of *Isocoma venetus* (Asteraceae, former genus *Haploppapus*) in California (Louda, 1982a, 1982b, 1983), as seed predation intensifies towards the coastal milder sites. Similar trends have been observed along elevational gradients across multiple taxa (Giménez‐Benavides et al., 2008; Kelly, 1998; Lord & Kelly, 1999; Molau et al., 1989; Randall, 1986; but see Vaupel & Matthies, 2012). Latitudinal studies on flower–insect visitor interaction that characterized cost–benefit ratios have yielded diverse results with some work showing an increase in pre‐dispersal seed predation with latitude (i.e., Chen et al., 2017) while other studies showing no latitudinal trends (Garcia et al., 2000; Moles & Westoby, 2003). Along elevational gradients, some studies analyzed the cost–benefit ratios in relation to pollinator abundance and activity (Cruden et al., 1976), or in relation to climatic gradients in the Yucca‐Yucca moth system (Harrower & Gilbert, 2018). In this later study, Harrower and Gilbert (2018) found that the ratio of fertile versus eaten seeds was lower at mid elevation sites than at sites situated at both extremes of the environmental gradient characterized by harsher environmental conditions. Such patterns have been attributed to the negative effects of harsher climatic conditions on insects and to smaller and less dense plant populations at the extremes of the climatic gradient (Hodkinson, 2005).

**FIGURE 1:**
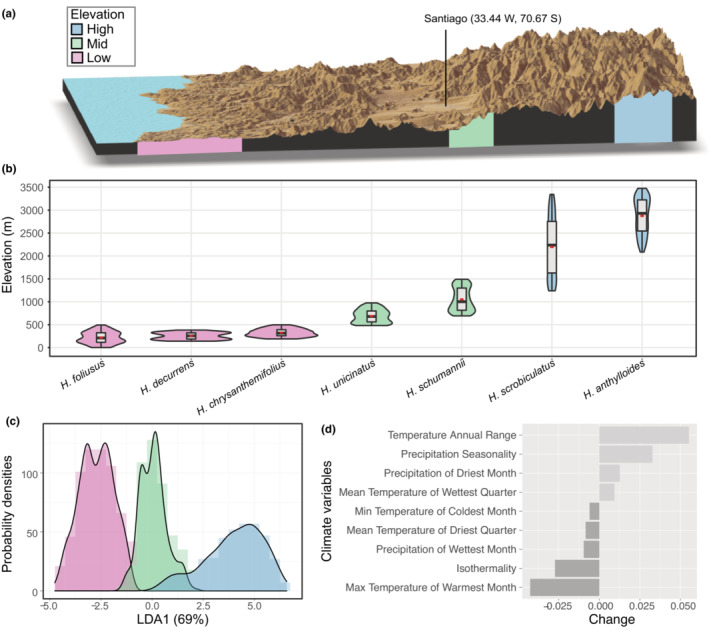
Elevational and climatic distribution of the seven *Haplopappus* species investigated. Shown are (a) the elevational profile of Central Chile, with highlighted the coastal, low elevation zone (pink shading), the inland mid elevation zone (green shading), and the Andean high elevation zone (blue shading); (b) Violin plots show the elevational distribution of the seven *Haplopappus* species, which were retrieved from Klingenberg (2007) (*n* = 500 randomly‐chosen spatial points per species); (c, d) Linear discriminant analysis (LDA) and change of climatic variables profiles along elevation gradients. (c) Histograms and density plots showing the distribution of discriminant scores of climatic profiles originating from the three elevation bands (low: pink shading, mid: green shading, and high: blue shading). The first LD1 explains 69% of the between‐group variance. (d) Discriminant coefficients for climatic variables included in the analysis. Variables with negative coefficients (in dark gray) reflect negative discriminant scores of the climatic niche (low‐elevation *Haplopappus* sp.), whereas compounds with positive coefficients (in light gray) reflect positive discriminant scores (high‐elevation species).

**FIGURE 2:**
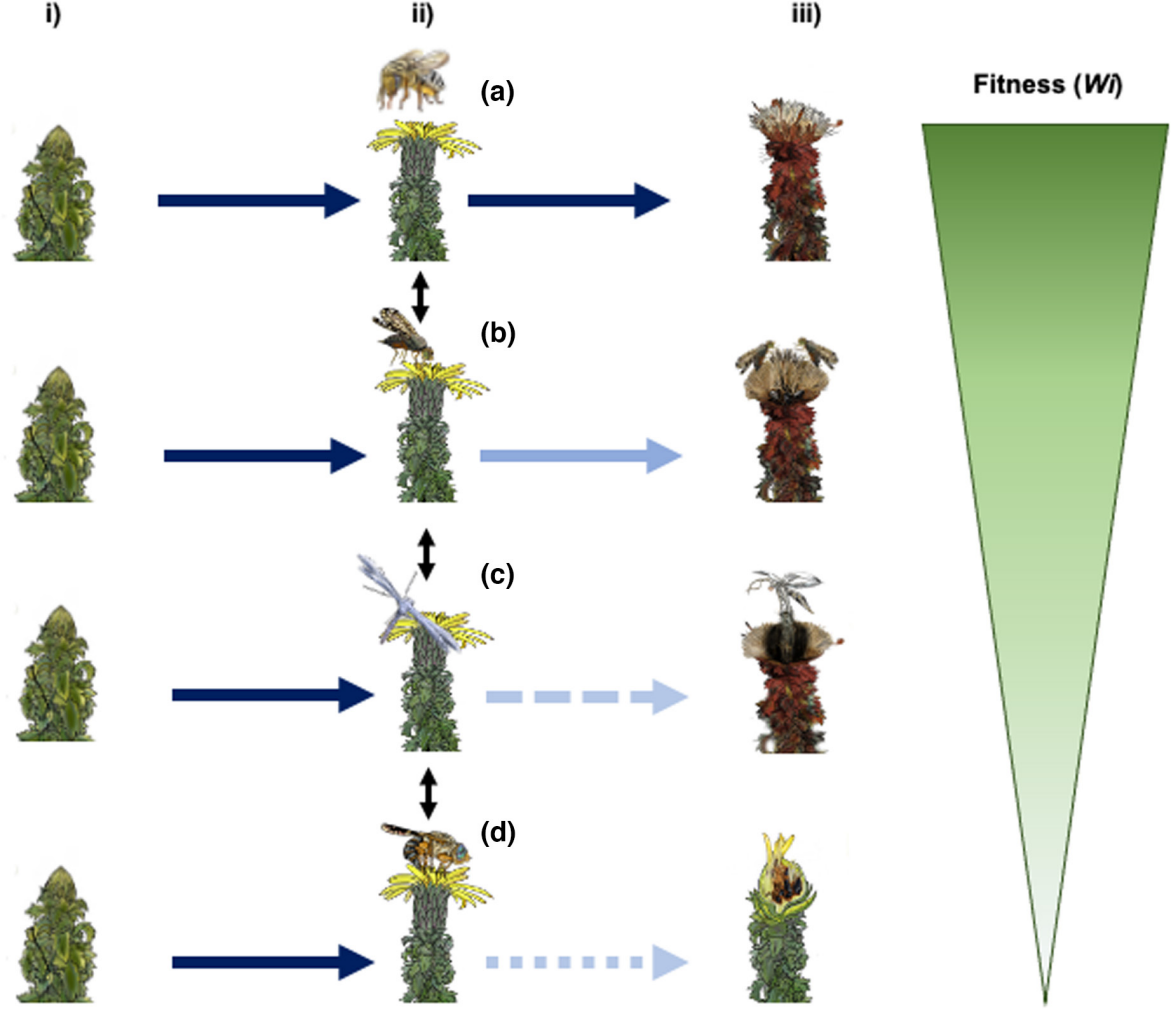
Overview of the *Haplopappus* pollination and seed predation system for three stages of the developing *Haplopappus* involucrum: (i) floral head bud (left); (ii) open floral disk when florets can receive visitors (middle); (iii) senescent capitulum with or without fruits (right). Four fates in decreasing order of fitness outcomes are depicted (top to bottom). In scenario (a) a floral head is visited by a legitimate pollen vector such as the native bee *Diadasia chilensis* (Apidae, Hymenoptera) resulting in seed production without subsequent seed damage. In scenario (b) the floral head is visited by the generalist fly *Dioxyna chilensis* (Tephritidae), which may allow some seeds to develop, either by previous legitimate pollinator visits or by incidental pollen deposition by this fly, while a few larvae will develop feeding on some ovaries and seeds. (c) A similar scenario is produced after the visit by microlepidoptera seed parasites, i.e., *Lioptilodes* sp. (Pterophorideae, Lepidoptera), nonetheless, floral head may lose a considerable part of the seeds. (d) Finally, if a floral disk is visited by an ovipositing *Trupanea* sp. (Tephritidae), there is little chance that any floret will produce seeds as most florets and ovaries are consumed by the developing larvae. Alternative fates are compared by a triangular bar to the right, highlighting possible fitness outcomes (*Wi*) resulting from the interaction with different visitors.

Here, we investigated patterns of floral fragrances and flower–insect visitor interaction along an elevation gradient. We asked whether congeneric plant species that had colonized different elevations had converged within their elevation zone on similar VOC blends and interactions with seed predators and potential pollinating insect visitors. We studied seven species in the genus *Haplopappus* Cass. (Asteraceae) that together cover the entire elevation gradient of Central Chile (Figure 1). In central Chile, the capitules of *Haplopappus* species host a variety of native insect visitors, including bees, flies, and lepidopteran moths (García et al., 2018; Villagra et al., 2021), which collectively constitute a diverse set of floral visitors (Savaris et al., 2015). Adult insects visiting *Haplopappus* shrubs feed on the florets while they rest and mate on the floral head disks (Frías, 2005). Females of Diptera and Lepidoptera species oviposit in the inflorescences and larvae feed on the developing ovaries and seeds (Figure 2; Villagra et al., 2014).

We asked the following questions: (1) Is there evidence for ecological convergence in VOC production among species within elevational bands? We expected floral odors to vary across species, but if they are evolutionarily labile, they should converge within elevation bands to optimize floral visitor repellence or recruitment according to the local arthropod community. The alternative hypothesis here is that convergence in flower odors is the product of phylogenetic convergence or inertia and variation correlates to relatedness. However, this cannot be tested due to a lack of phylogenetic information in this genus. (2) Does species variation in VOC relate to differential insect visitation? Considering that morphological floral head traits are highly comparable across *Haplopappus* species (Klingenberg, 2007), we hypothesized that it is the variation in floral fragrances that is involved in shaping diversification in *Haplopappus* insect recruitment. In other words, we expected that the observed differences in flower VOCs across species and sites to be functionally relevant, in particular in relation to major insect visitor species (Vargas et al., 2018). Accordingly, because lower‐elevation sites bear the highest abundance and diversity of insects, we expected low‐elevation *Haplopappus* species to be more attractive to floral visitors than high‐elevation species. (3) Finally, we asked whether there is ecological convergence within elevation bands in the outcome of interactions with seed predators and pollinators, quantified as the ratio between seed predation and seed production? A decline in the number of insect visitors was previously observed from low to high elevation (Vargas et al., 2018), likely due to the characteristic drastic changes in climatic conditions along the Andean elevational gradients (Luebert & Pliscoff, 2006). Based on this, we expected that the cost–benefit ratio will increase with elevation. Altogether, we predict that VOC production is labile, with species within the same elevational bands converging on similar fragrances and having similar interactions with floral visitors.

## 2 METHODS

### 2.1 Study system

The *Haplopappus* (Asteraceae) genus represents a group of perennial shrub species native to the Andes, including within its distribution range, parts of Peru, Bolivia, and Chile. In Chile, *Haplopappus* is one of the most abundant floristic elements in the arid and semiarid part of this country, including over 60 plant species that together span the Pacific coastal terraces to the alpine habitats of the Andes, up to 4000 m above sea level (Brown & Clark, 1981; García et al., 2018; Klingenberg, 2007; Moreira‐Muñoz, 2011). To address our aims, we selected seven species of *Haplopappus* from Central Chile, occurring at three distinct elevation bands, with little or no populational overlap (Klingenberg, 2007), from the coastal area of Chile up to the limit of vegetation in the Andes (Figure 1a). From low elevation sites, Los Molles, Petorca Province V Region, (20 meters above sea level (m a.s.l.), 32°14′22″ S 71°30′54″ W, and Quintay, Valparaíso Province V Region (69 m a.s.l.), 33°11′00″ S 71°41′10″ W, we sampled *H. foliosus*, *H. chrysanthemifolius*, and *H. decurrens* shrubs. From mid‐elevation sites: Yerba Loca (1415 m a.s.l.), 33°20′00″ S 70°18′00″ W, and El Volcán (1325 m a.s.l.), 33°48′42″ S 70°12′22.7″ W, Cordillera Province, we sampled *H. velutinus* and *H. uncinatus*. From high elevation sites, Juncal (2771 m a.s.l.), Farellones (2532 m a.s.l.), 33°20′59″ S 70°18′34″ W and Termas Colina (2631 m a.s.l.), 33°51′04.59″ S 69°59′35.19″ W, Cordillera Province, we sampled *H. anthylloides* and *H. scrobiculatus* (Figure 1b).

### 2.2 Extraction of climatic variables

To characterize the climatic niche of each *Haplopappus* species, we first georeferenced and rasterized the distribution map as described in Klingenberg (2007) (Figure S1). From the geotiffs of each species, we randomly extracted 500 geographic locations and removed the upper and lower 10th quantiles of extreme elevations. We then extracted the 19 BIOCLIM variables from the Chelsa global climate data set (https://chelsa‐climate.org/bioclim/) at 30‐s resolution (Karger et al., 2017) from the remaining georeferenced points. For statistical analyses, we removed overly‐correlated variables from the full list using the package *caret* (Kuhn, 2008) in R (R Core Team, 2020) resulting in nine uncorrelated variables describing the climatic niche of each elevation band of Central Chile. We further visualized the average climate across the three elevation bands (low, mid, and high elevation) across all species using linear discriminant analysis on the climatic data matrix (*lda* function in the MASS package v. 7.3‐54; Venables & Ripley, 2002), and visualized the ordination for each elevation band using the first axis of the LDA. To assess differences in climate across elevation bands, we performed a Distance‐Based Redundancy analysis (dbRDA) of the climatic data based on Euclidean distances (*capscale* function in *vegan 2.6‐2*; Oksanen et al., 2013), and assessed the significance using a permutation test. Not surprisingly, we could highlight a clear differentiation in the climatic niche across the three elevation bands (ANOVA based on 999 permutations, *F*_1,1397_ = 2753.5, *p* < .001). Particularly, low‐elevation coastal sites were characterized by a warm and highly isothermal climate, but also by more precipitation than at the other two elevations. Conversely, the high‐elevation Andean sites were characterized by colder and drier climates, as well as high precipitation seasonality and the highest amplitude in annual temperature (Figure 1c,d).

### 2.3 Natural history of *Haplopappus*–insect interaction

While natural history observations show that the flowers of most *Haplopappus* species sampled host a variety of insect visitors, including potential pollinators (i.e., syrphid flies, bees), and seed predators (see below), the dependence of these plants on pollinator visitation remains largely unknown. What is clear however, is that the ovaries and seeds of *Haplopappus* shrubs are known to be attacked by several insect species, including by *Lioptilodes* (Pterophoridae) micromoth seed predators (Vargas et al., 2018; Vargas & Parra, 2005) or by tephritid flies from the genus *Trupanea* (Frías, 2005) and *Dioxyna* (Frías, 2005; Vargas & Parra, 2005; Villagra et al., 2014). Different seed predators found in these plants also display varying degrees of specialization. For instance, floral heads (or capitula, used interchangeably in this paper) of *H. foliosus* are mostly parasitized by the specialist *Trupanea foliosi*, while the sympatric *H. decurrens* is mostly attacked by *Trupanea simpatrica*. These flies oviposit in the early stages of floral head buds and larvae feed on developing seeds, even stunting floral head development (Figure 2; Frías, 2005). Moreover, the micromoth *Lioptilodes friasi* (Pterophoridae) seems to be specialized at the genus level (Vargas et al., 2018; Villagra et al., 2014). Finally, several *Haplopappus* species can be attacked by the generalist seed predator *Dioxyna chilensis*. Nonetheless, this relatively small fly exerts less damage to inflorescences than the more specialized *Trupanea* species, since in Chile *D. chilensis* is also able to oviposit on alternative Asteraceae hosts (Frias, 1992). Previous natural history observations on this system have highlighted that while the relative abundance of insect visitors changes across species, from low to high elevation, the most common insect visitors (i.e., the tephritid flies: *T. chilensis* and *D. chilensis*) are broadly distributed across the range (Vargas et al., 2018). This suggests that the *Haplopappus* plant clade, while it radiated across the elevation gradient into different habitats, it conserved its association with a taxonomically very similar guild of floral visitors.

### 2.4 Floral volatile organic compounds sampling and analysis

To analyze differences in flower VOC production across elevational bands, we sampled the headspace of single floral heads (or capitula) of natural populations of *Haplopappus* species occurring at three distinct elevational zones (two to three species per elevational band, *n* = 5–10), using polydimethylsiloxane (PDMS)‐coated Twisters (Gerstel, 10 mm length, 0.5 mm film thickness). Sampling occurred in January 2018, between 11 AM and 2 PM, on sunny days. For each *Haplopappus* species, we randomly selected one floral head per plant with florets in the floral disk in full bloom and bagged it using 1 L oven bags (Tangan No34, Turkey). Next, the headspace samples were pumped on the Twisters with a handpump set to 200 mL/min. The pump was connected to a glass tube within which we inserted the adsorptive Twister, which was inserted into the bag and allowed to collect for 2 h. Additionally, we collected control samples (VOCs collected from the vegetative part of the plant but without the flowers inside the bag) using the same methodology as the floral samples. The hermetically sealed Twisters tubes were stored in a cooler in the field and then stored in a freezer at −20°C within the same day, where they remained frozen until gas chromatography (GC) analysis. After including 1 μL internal standard (5 μg/mL naphthalene in dichloromethane) directly to each Volatile compounds were thermally desorbed using a Multipurpose Sampler MPS (Gerstel), and injected onto an HP5MS column, 30 m × 0.25 mm × 0.25 μm at 40°C for 30 s. For VOCs separation, the GC oven temperature was increased at 5°C per minute until 160°C, which was held for 0.01 min before increasing the temperature again at 3°C per minute until 200°C, which was held for 4 min before a final temperature ramp at 100°C per minute until reaching 250°C for 3 min. VOCs were then detected by within an Agilent 5975C (Agilent) mass spectrometer (MS). The mass detector in EI mode at 70 eV was used to scan over the mass range from m/z 33 to 350. To identify the molecules, we compared each peak found in the chromatograms with the NIST05 database. Compounds present in control samples were removed from our final analysis. Tentative compound identification was done using AMDIS and NIST libraries, as well as with comparisons with in‐house libraries of mono‐ and sesquiterpenes mixtures. All compounds detected in each *Haplopappus* species are reported in Carvajal Acosta et al. (2023).

**FIGURE 3:**
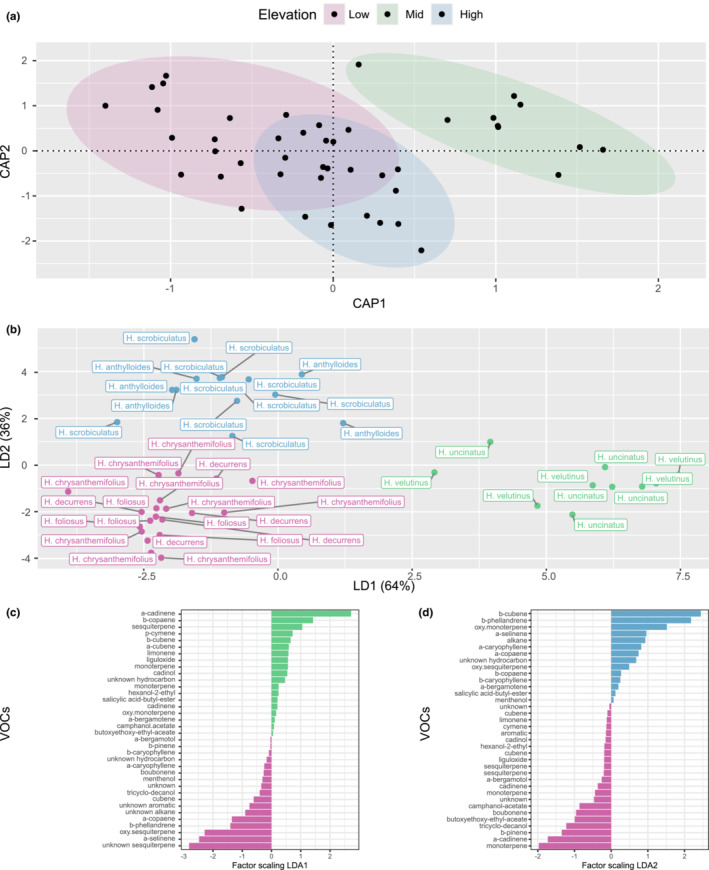
Linear discriminant analysis (LDA) and change of *Haplopappus* spp. flower volatile profiles along elevation gradients. (a) Distance‐based Redundancy Analysis (dbRDA, capscale) plot derived from Kulczynski dissimilarities of the VOCs composition sampled from species of *Haplopappus* belonging to low (pink ellipses), mid (green ellipses) and high (blue ellipses) elevation bands. Effect of elevation (*F*_2,42_ = 2.07, *p* = .008), and axes effect (CAP1; *F*_1,42_ = 2.53, *p* = .017, and CAP2; *F*_1,42_ = 1.62, *p* = .08). (b) LDA biplot distribution of discriminant scores of flower volatile organic compounds (VOCs) profiles released by the different *Haplopappus* spp. originating from different elevations bands as shown in Figure 2a (low elevation zone (pink shading), the inland mid elevation zone (green shading), and the Andean high elevation zone (blue shading). (c) Discriminant coefficients for each compound included in the overall volatile blend along the first axis of the LDA, and (d) along the second axis of the LDA. Compounds with negative coefficients (in pink) reflect negative discriminant scores of flower volatile profiles of low‐elevation species, whereas compounds with positive coefficients in green for (b), and in blue for (c), reflect positive discriminant scores for mid‐ and high‐elevation species, respectively. VOC names were received by comparison with of library spectral structures, and annotations should be considered tentative.

To assess differences in VOCs across elevation bands, we performed a Distance‐based redundancy analysis (dbRDA) after Pareto‐transformation of the data and based on Kulczynski distance (*capscale* function in *vegan* v.2.6‐2; Oksanen et al., 2013). We visualized the clusters of species across the three elevation bands (low, mid, and high elevation) using first the results of the dbRDA analysis, and then, for better distinguish VOCs discrimination, we used a linear discriminant analysis on the VOC data matrix (*lda* function in the MASS package v. 7.3‐54; Venables & Ripley, 2002), after testing for homogeneity of multivariate dispersion with the function *betadisper* in *vegan* v.2.6‐2 (Oksanen et al., 2013) (*F*_2,42_ on 99 permutations = 1.73, *p* = .17; Figure 3).

### 2.5 Insect preference bioassay

To investigate whether floral heads from different elevation bands were differentially attractive to floral visitors, we measure individual preferences from the most common insect visitors, *Dioxyna chilensis* and from *Trupanea* sp. seed predators, towards *Haplopappus* floral heads growing at the two extremes of the elevation gradient (i.e., low‐ and high‐elevation species). Both fly species have been described in Soto Andrades (2021). We constructed seven arenas by taping a BugDorm mesh lid to the bottom of an inverted a plastic pot (Figure S2). We inserted a pair of inflorescences in each arena with *H. foliosus* from low elevation and *H. scrobiculatus* from high elevation. *D. chilensis* and *Trupanea* sp. flies emerging from inflorescences collected from all *Haplopappus* species along the elevation gradient were used for the choice bioassay. Each bioassay consisted of placing a pair of flies, a male and a female of the same species, in an arena for 10 min and counting the number of landings on each inflorescence (*n* = 30 pairs for *D. chilensis,* and 20 pairs for *Trupanea* sp.). Flies were introduced in pairs to incentivize them to make a choice as both fly species mate in the flowers. Females were identified based on the presence of an ovipositor. We recorded the number of landings throughout the duration of the bioassay and reported this data in Carvajal Acosta et al. (2023). In a few instances, one or both flies landed on a flower once for the entire duration of the bioassay. We converted landing counts to proportions so that a fly that could not decide between flowers would not outweigh a fly who made a clear choice from the start. Proportions of landing were estimated by dividing the number of landings on each floral head by the total number of landings and this data were reported in Table S2. To test whether flies prefer high or low‐elevation capitulum, we performed a generalized linear model (glm), with a floral head elevation of origin (high or low) as a fixed factor and using a quasibinomial distribution. Statistical differences were quantified using the function *ANOVA* in the package *car* (Fox & Weisberg, 2012). Because there were no obvious sex differences in landing choice, we grouped male and female choices for the analyses, but *D. chilensis* and *Trupanea* sp. datasets were analyzed separately. A first model that included cage as a blocking factor showed no effect of cage (for *Dioxyna*; $\chi_{6}^{2}$ = 12.17, *p* = .06, and for *Trupanea*; $\chi_{4}^{2}$ = 3.09, *p* = .54), and was thus removed from the final model. Moreover, all adults of both species were kept together, separated by species, in common rearing cages till the bioassay, and thus we were not able to track back the origin of the individual flies for this analysis.

### 2.6 Predation versus fertilized seed counts across species

To assess the potential positive or negative effects of floral visitors, we randomly sampled 10 capitula that were at the mature stage across approximately 20 plants for each *Haplopappus* species (*n* = 18–34 plants per species, with a total of ~1500 capitula). Capitula were placed individually in plastic tubes with pierced lids, so to let them fully mature under controlled conditions in the laboratory. After a minimum of 2 weeks, each capitulum was analyzed under a stereo microscope for quantifying fertile (fully swollen) seed production, as well as the number of seeds that were visibly damaged by an herbivory event. In most cases, a small exit hole can be observed in the seed, meaning that herbivory by an insect larva had happened inside the seed (Figure 2). Seed herbivory and fertilization data can be found in Carvajal et al. (2023). We opted to not quantify unfertile seeds since we could not assess the fertility of half‐swollen seeds. We also documented the taxonomic identity and abundance of insects emerging from each flower head (Figure S3). To estimate the effect of elevation on the number of herbivore‐damaged and fertilized seeds, we performed a generalized linear model (glm) with a quasi‐Poisson error structure. Statistical differences were quantified using the function *ANOVA* in the package *car* (Fox & Weisberg, 2012). To estimate the likelihood of benefits versus costs imposed by floral visitors on plants, we calculated, for each *Haplopappus* species, an effect size between the fertile seeds and the herbivore‐damaged seeds. Effect sizes were calculated using Cohen's *d* metric (Cohen, 1988), as estimated with the *effsize* package v. 0.8.1 (Torchiano, 2020). In our case, higher, positive, effect sizes indicate more costs (incurred by being eaten by seed predators) than benefits (incurred by being pollinated).

## 3 RESULTS

### 3.1 Volatile organic compounds

Based on our GC–MS analysis, across species, we retained 34 VOCs that were present in at least two individual plants, including mostly sesquiterpenes (23), and monoterpenes (11) in different proportions across species (Table S1; Figure 3). We found clustering of VOC blends based on elevation bands (Figure 3a,b), ANOVA based on 999 permutations, *F*_2,42_ = 2.07, *p* < .005; *R* ^2^ values for species are .22 and .1 for elevation). Specifically, we found that *Haplopappus* shrub species growing at low and mid‐elevation clearly separated along the first axis of the LDA (Figure 3c), while the high‐elevation VOCs profiles were mainly separated along the second axis of the LDA (Figure 3d). The separation was strongly driven by unique blends of VOCs, mainly composed of different mono and sesquiterpenes (see details in Figure 3).

### 3.2 Insect preference bioassay

**FIGURE 4:**
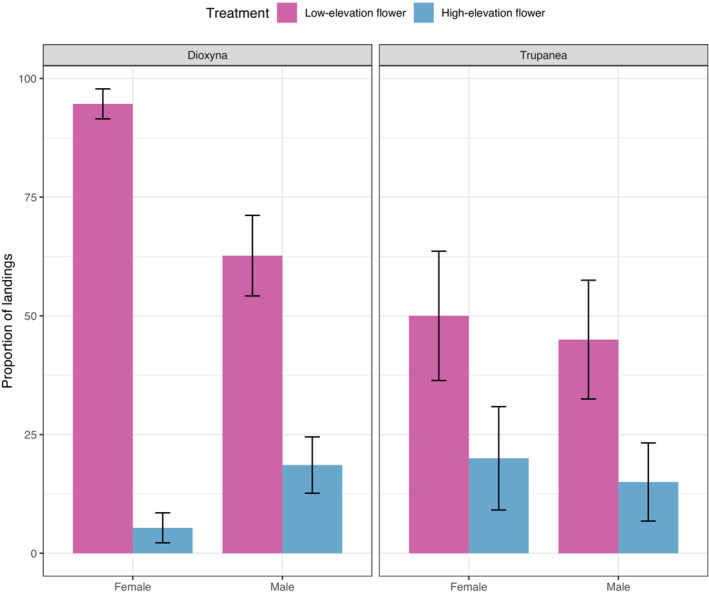
Insect preference bioassay. Barplots showing average proportions of landings on floral disks of the high‐elevation *Haplopappus scrobiculatus* plants (blue bars), or of the low‐elevation *H. foliosus* plants (pink bars). Error bars represent standard deviations. Two tephritid fly species were tested, *Dioxyna* sp., shown on the left panel, and *Trupanea* sp. shown on the right panel. The graph shows the proportions for both females and male flies.

We found that both *Trupanea* sp. and *D. chilensis* flies preferred landing on the low‐elevation species (*H. foliosus*) than on the high‐elevation species (*H. scrobiculatus*) (Figure 4; for *D. chilensis*: Type II analysis of Deviance, LR = 45.07, df = 1, *p* < .001, and for *Trupanea* sp. LR = 4.45, df = 1, *p* < .03). Specifically, the probability of landing on the low‐elevation flower head was 6.5 and 2.1 times higher for *D. chilensis* and *Trupanea* sp., respectively, than on high‐elevation capitula.

### 3.3 Seed predation to fertile seeds ratios across elevation bands

**FIGURE 5:**
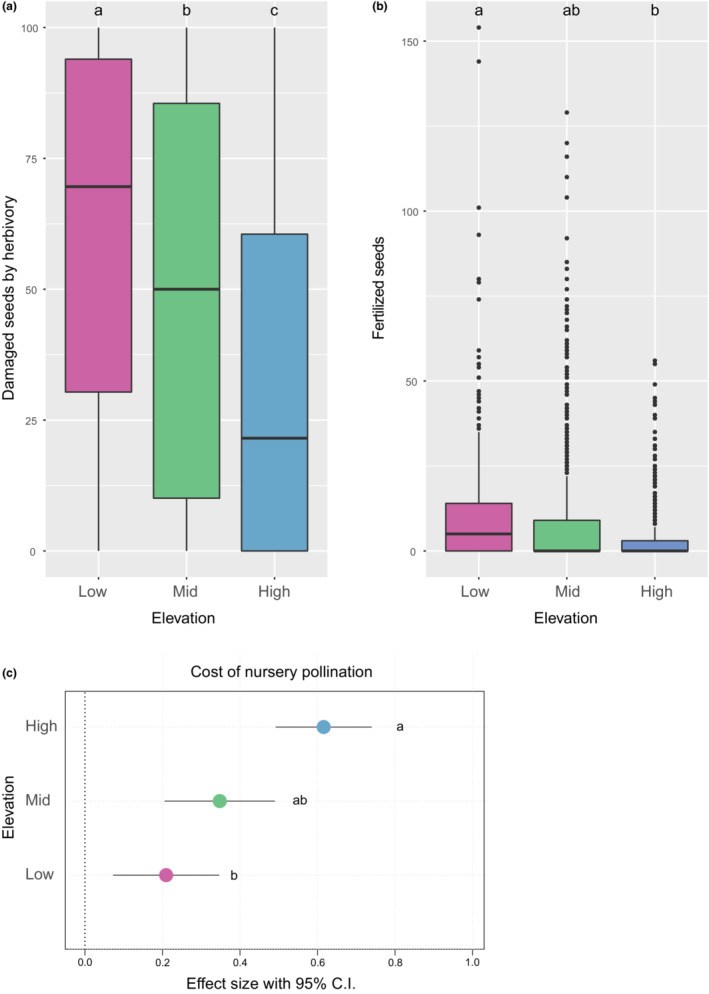
Seed predation to fertile seeds ratios across elevation bands. Shown are (a) boxplots depicting the number of damaged seeds per plant found across the three elevation bands as shown in Figure 2a (low elevation zone (pink shading), the inland mid elevation zone (green shading), and the Andean high elevation zone (blue shading). (b) The number of fertile seeds across the three elevation bands (*n* = 15–68 plants per species, and 2–9 floral heads per plant, for a total of 1505 flower head measured). Panel (c) shows the effect size (Cohen's *d*) between fertile and damaged seeds across the three elevation bands. Positive values indicate an over‐representation of damaged seeds compared to fertile seeds. Letters show differences across elevation bands based on Tukey HSD post hoc test (panels a and b) and based on 95% confidence intervals (C.I.) (panel c).

We found that both the number of fertile seeds (Type II analysis of deviance, LR = 54.25, df = 2, *p* < .001), and the number of damaged seeds (Type II analysis of deviance, LR = 145.74, df = 2, *p* < .001) decreased with elevation. On average the number of fertile seeds was 2.4 times higher, and the number of damaged seeds was 1.85 higher on low‐elevation sites compared to high‐elevation sites (Figure 5a,b). We found that, across the three elevations, the ratio of damaged seeds to fertile seeds was 5.8:1, for every fertilized seed about six were damaged (Figure 5a,b). However, the relative predation rate increased with elevation; at high elevation sites, we observed that for eight damaged seeds one was pollinated, compared to a 5:1 ratio at mid‐elevation, and a 6:1 ratio at low elevation sites, where for every six damaged seeds one was fertilized (Figure 5c). Particularly, we observed the relative abundances of seed predators were higher at low‐ and mid‐elevations with *Liptilodes* spp. and *Dyoxina* spp. being the most abundant. While at high elevations, *Trupanea* flies were the most abundant seed predators, which was particularly higher in *H. anthylloides* flowers (Figure S3).

## 4 DISCUSSION

Convergence in plant traits could be the result of multiple ecological and evolutionary factors. Thus, plants should emit similar VOC blends in response to ecological factors such as local abiotic (Holopainen & Gershenzon, 2010), biotic conditions (Penuelas & Llusià, 2001) or it might be simply driven by phylogenetic inertia (Kergunteuil et al., 2020). Here, we tested whether variation in floral odors and flower–insect visitor interaction along elevation gradients have converged for *Haplopappus* genus occurring within the same elevation bands. We found convergence within elevation bands in both floral scent and in the outcome of biotic interactions for inflorescences, assessed as the ratio of seeds predated to ovules fertilized. Furthermore, plants from contrasting elevations were differentially attractive to common flower visitors, suggesting that the measured differences in flower VOCs are indeed functionally active for flower visitors. Together, these results suggest that radiation to differing elevational zones in this plant clade resulted in convergence of species traits with consequences for the resultant ecological interactions.

Convergence of floral traits such as morphology, color, and scent in response to similar floral visitor functional groups (i.e., seed predators and potential pollinators) have been observed in several plant–insect associations (Fenster et al., 2004). Accordingly, we proposed that *Haplopappus* species have converged to produce similar fragrance bouquets within elevation bands to optimize insect visitor recruitment according to their local community. In accordance with this prediction, *Haplopappus* VOC profiles clustered with elevation, such that *Haplopappus* species within the same elevation bands produce more similar VOC bouquets than across different elevations. We speculate that differences in VOC blends were driven by the distinct patterns of insect diversity among sites (Figure S3). Interestingly, we observed different distributional patterns in the tephritid flies studied than in a previous survey of *Haploppapus* spp. While Vargas et al. (2008) found that both fly species were broadly distributed along the gradient, we observed that *Trupanea* sp. emerged almost exclusively from high‐elevation sites whereas *Dioxyna* sp. was more abundant at low‐elevation sites. These trends were evident in previously collected flower head samples (Figure S3) as well as in flower heads collected for the choice bioassay. Although the entire floral visitor community is unknown, seed predator diversity is generally higher in low and mid‐elevation sites compared to high‐elevation sites (Figure S3). Thus, low‐elevation species may produce volatiles that are more repellent to seed predators, but this explanation is unlikely as both fly species preferred low‐elevation flower heads. Alternatively, at high elevations, where insect abundance is the lowest, plants may rely more on wind rather than insects for pollination (Damialis et al., 2011). Therefore, it is possible that high‐elevation *Haplopappus* species are under less selective pressure to produce volatiles attractive to pollinators. Evolutionary convergence for floral volatiles has also been observed in nursery pollination systems, in which the same insect acts as pollinator and seed predator (i.e., *Lithophragma*–*Greya*; Friberg et al., 2019). In the case of the *Lithophragma*–*Greya* system, these evolutionary patterns are believed to have evolved in response to the geographically diverse interactions with specialist and generalized co‐pollinators. However, other studies examining geographic variation in VOCs in other plant–insect interactions have not shown variation in floral volatile composition. For example, Svensson et al. (2005) investigated interpopulation variation in Yucca trees VOC emissions demonstrating that the floral scents were similar among plant populations even when floral visitors were different. In another study involving *Trollius* (Ranunculaceae) and its pollinating flies, Ibanez et al. (2010) also found some geographical variation in floral scent, but with large overlap in scent samples among the populations. Therefore, while convergence in phytochemical diversity in response to local biotic conditions seems to be widespread, it is not the rule.

In addition to selection by insect visitors, patterns of ecological convergence in VOCs within elevation bands could also be partly driven by contrasting climatic conditions (i.e., different temperatures and humidity regimes would impose different emission rates on VOCs; Holopainen & Gershenzon, 2010). Low‐ and high‐elevation *Haplopappus* species emitted distinct monoterpenes and sesquiterpenes compounds at higher rates (Figure 3). These compounds may play an important role in how plants cope with harsh environmental conditions along the gradient. For example, monoterpenes concentrations in *Artemisia brevifolia* increase with elevation (Nataraj et al., 2022) and have been previously suggested to play a role in plant's acclimation to high UV radiation (Nikolić et al., 2011). Similarly, certain monoterpenes, namely eucalyptol, camphor, and alpha/beta‐thujone accumulate under drought stress (Nowak et al., 2010) which tends to be a common stressor at high‐elevation sites of the Chilean Andes. Moreover, severe environmental conditions may simply preclude plants from producing compounds below certain temperature or humidity thresholds (Duhl et al., 2008). Finally, climate may also influence convergence in floral VOCs via phenotypic plasticity (Pigliucci et al., 2006). While VOCs have been found to not be particularly plastic in some systems (Friberg et al., 2017; Luizzi et al., 2021), phenotypic plasticity appears to play a major role in others (de Manincor et al., 2022). However, because *Haplopappus* species occurring at different elevational bands have distinct non‐overlapping ranges with their unique climatic niches, whether VOCs convergence is the result of phenotypic plasticity cannot be ascertained without experimentally exposing the same species to a range of climatic conditions (i.e., reciprocal transplants). Consequently, changing abiotic conditions from low to high elevation may have also contributed to the observed convergence patterns across species growing in similar habitats but the specific mechanisms (i.e., physiological stress or constraints, phenotypic plasticity) need to be further explored.

That said, with our system, we cannot fully exclude that similarities in floral odors could also be partly driven by phylogenetic inertia. Unfortunately, we were unable to test this hypothesis as, to date, gene sequences for building a proper phylogenetic tree of the group are virtually absent – despite our attempts to extract DNA from these chemically complex plants. However, based on evidence from other systems, we suspect that phylogenetic inertia plays a minor role in VOC convergence. For instance, observed patterns of phytochemical convergence along elevation gradients have been found to be independent of phylogeny (Bakhtiari et al., 2021; Defossez et al., 2021; Eisen et al., 2022; Pearse & Hipp, 2012), while instead we mostly observe patterns of chemical divergence among sites (Becerra et al., 2009; Kursar et al., 2009; Salazar et al., 2018). Moreover, in the *Haplopappus* system, evidence from a morphological traits‐based cladogram (Klingenberg, 2007) suggests that the species studied here do not belong to the same clade. Thus, phylogenetic inertia may be a less important factor driving VOCs convergence in *Haplopappus* species within sites than factors such as climate or biotic conditions. Yet, until a phylogenetically controlled analysis is performed, whether *Haplopappus* species produce similar VOC blends within the same elevation bands due to shared ancestry remains to be ascertained.

In line with variation in floral odors across elevations, we found that both fly species tested preferred flower heads from the low‐elevation *H. foliosus* over flower heads from the high‐elevation *H. scrobiculatus*. Although the elevational origin of the flies was not included in our analysis, as mentioned previously, we noticed that *Trupanea* sp. emerged almost exclusively from high‐elevation flower heads while *Dioxyna* sp. from low‐elevation sites. It is interesting to note that both fly species consistently preferred the low‐elevation *Haploppapus* flower heads despite of their differences in elevational ranges and the sex of the fly. Because floral morphology is quite consistent among *Haplopappus* species (Klingenberg, 2007), the results suggest that these flies may indeed use floral head VOCs for locating their preferred host plant as in other plant–insect interaction systems (Hossaert‐McKey et al., 2010). Terpenes are emitted by many plant species, and they have also been identified as the main compound classes responsible for the attraction of insects in various nursery pollination interactions, including *Elachipetra formosa* flies (Diptera: Chloropidae) and their host plant *Peltandra virginica* (Araceae; Patt et al., 1992), the tightly coevolved fig‐fig wasp system (Soler et al., 2011; Souto‐Vilarós et al., 2018) as well as the Yucca‐Yucca moth system (Svensson et al., 2005). Thus, it is possible that unique blends of monoterpenes and sesquiterpenes identified in our analysis are involved in the attraction of *Tephritidae* flies to *Haplopappus* inflorescences. Of course, one limitation of our study is that we used one representative species from low and high‐elevation bands. However, as we discussed above, VOC profiles were similar among co‐occurring *Haplopappus* species along the altitudinal gradient; thus, we suspect that adding more species would yield similar results. Nonetheless, as postulated in the introduction, *H. foliosus*, being abundant near the coastline of Central Chile and growing over extensive areas of the landscape (Klingenberg, 2007), might have found the optimal volatile blend that is highly attractive to secure the necessary reproduction requirements, while proportionally diluting the costs incurred by seed predation on few individuals.

In general, our findings are in line with past work showing that pre‐dispersal seed predation decreases with elevation (Giménez‐Benavides et al., 2008; Kelly, 1998; Lord & Kelly, 1999; Molau et al., 1989; Randall, 1986). This pattern is thought to arise from a general degradation of the climatic conditions at higher elevations, including colder temperatures, higher temperature oscillations, drier conditions, and shorter growing seasons, altogether inhibiting the activity of seed predators (Hodkinson, 2005). Based on these trends, we then expected the cost–benefit balance to be higher at sites with milder environmental conditions where seed predation is higher. In contrast, we found a progressive increase in the cost–benefit ratio with elevation, peaking at the highest elevation zones (i.e., in the alpine environments). In this regard, our results also differ from those observed in the yucca‐yucca moth system, in which both seed predation and fertilization rates were higher at mid‐elevation sites, where climatic conditions were the mildest (Harrower & Gilbert, 2018).

There are several possible explanations for the observed cost–benefit ratio trends across the gradient. Among the most likely explanations is that these patterns are driven by variation in resource abundance. Because plants and flowers are more abundant at mild low‐elevation bands, the per capita cost of attracting more seed predators is diluted whereas the per capita cost of seed predation increases in alpine habitats, where plants are sparser (Rasmann et al., 2018). Second, it is also possible that floral visitor abundance may influence the cost–benefit balance equally or more strongly than the resources represented by the host plants. For instance, in the yucca‐yucca moth cost–benefit analysis, the cost–benefit ratio was higher at mid‐elevation sites, where moth abundance was also higher, this despite the higher abundance of host plants and flowers at these sites (Harrower & Gilbert, 2018). Another example highlighting the importance of pollinator presence is the *Greya‐Lithophragma* nursery pollination system. This interaction is generally mutualistic, however, the net outcome may shift to antagonism, depending on the number of generalized co‐pollinators present (Thompson & Cunningham, 2002). In our case, floral visitor abundance was higher at lower elevations and decreased with elevation along the gradient (Figure S3). Thus, plants could also be pollinated at higher rates by co‐pollinators, including legitimate pollinators, thus diminishing the negative effect of seed predation by *Tephritidae* flies. Indeed, although *Tephritidae* flies occur across the entire elevational gradient, Tortricidae moths are the co‐dominant seed predator, along with *Dioxyna*, at low elevations. In particular, *Lioptilodes* moths are more abundant at low‐elevation sites and these insects tend to consume fewer seeds than *Tephritidae* flies (Frias, 1992). Future manipulative studies should specifically address the pollination success of the main floral visitors on *Haplopappus,* including nocturnal pollinators, as well as any evolved mechanisms for regulating seed predation cost, including estimating the role of predators and parasitoids attraction for regulating herbivore densities.

In summary, whether the convergence of floral odors is driven by climatic constraints, phylogenetic inertia, or whether floral VOCs are more under biotic selection to maximize pollinator attraction versus predator repulsion at each site, needs to be further teased apart, by for example performing reciprocal transplant plant experiments across different elevations. Nonetheless, our results suggest that patterns of ecological convergence in phytochemistry may be a major driver of biotic interactions and their subsequent fitness outcomes in *Haplopappus* species along an elevational gradient. This study represents a step forward toward understanding the factors driving the ecological convergence of floral scent and their consequences for conditional outcomes in plant–insect interactions. However, there is still much to learn about the evolutionary, ecological, and environmental factors influencing the evolution of floral volatiles. Furthermore, climate change may alter the conditions in which these tightly co‐evolved associations occur in many different ways, for example, by prompting an increase of pollinators or seed predator insects at higher elevations (Inouye, 2020; Marshall et al., 2020) or by changing volatiles blends used as locating cues for pollinators and pre‐dispersal seed predators (Farré‐Armengol et al., 2014; Rering et al., 2020). The findings from this study will allow us to further our understanding of the context‐dependency driving tightly linked biotic interactions and improve our understanding of how plant and insect communities might be reshuffled due to global climate change (Descombes et al., 2020). Nonetheless, further research is needed for building a theoretical framework that can accurately predict biotic interaction outcomes under future climatic conditions.

## AUTHOR CONTRIBUTIONS

**Alma Nalleli Carvajal Acosta:** Conceptualization (equal); data curation (equal); formal analysis (supporting); investigation (lead); methodology (equal); writing – original draft (lead); writing – review and editing (lead). **Ludovico Formenti:** Conceptualization (supporting); methodology (equal); writing – review and editing (equal). **Adrienne Godschalx:** Investigation (equal); methodology (equal); writing – original draft (supporting); writing – review and editing (equal). **Cristian Villagra:** Conceptualization (supporting); investigation (equal); visualization (equal); writing – original draft (equal); writing – review and editing (equal). **Kailen Mooney:** Conceptualization (equal); methodology (supporting); supervision (supporting); writing – review and editing (equal). **Angelos Katsanis:** Methodology (equal); writing – original draft (supporting); writing – review and editing (equal). **Constanza Schapheer:** Methodology (equal); writing – review and editing (equal). **Sergio Rasmann:** Conceptualization (equal); formal analysis (lead); funding acquisition (lead); investigation (equal); methodology (equal); project administration (lead); resources (lead); supervision (lead); visualization (lead); writing – original draft (supporting); writing – review and editing (equal).

## Supporting information


Appendix S1

## Data Availability

All data is publicly available in the supplemental materials and in Dryad at: https://doi.org/10.7280/D1RT3J.
