# Supplementary material for: Ecological convergence in phytochemistry and flower–insect visitor interactions along an Andean elevation gradient
Source: Ecol Evol. 2023 Aug 16;13(8):e10418. doi: 10.1002/ece3.10418 (PMC10432872; doi:10.1002/ece3.10418)
Supplement: Supplementary file 1 — Appendix S1 [file ECE3-13-e10418-s001.docx]

Supplementary material for:

**Ecological convergence in phytochemistry and plant-flower visitor interactions along an Andean elevation gradient**

A. Nalleli Carvajal-Acosta, Ludovico Formenti, Adrienne Godschalx, Angelos Katsanis, Kailen Mooney, Constanza Schapheer, Cristian Villagra, Sergio Rasmann

**Tables**

**Table S1.** Volatile organic compounds (VOCs) emitted by *Haplopappus* flowers. Shown are the identified compounds (marked in bold and with an asterisk) based on in-house compound libraries of pure compounds, and the tentative identification based on the NIST VOCs library across seven *Haplopappus* species.

| VOCs | *H. anthylloides* | *H. chrysanthemifolius* | *H. decurrens* | *H. foliosus* | *H. scrobiculatus* | *H. uncinatus* | *H. velutinus* |
| --- | --- | --- | --- | --- | --- | --- | --- |
| b-phellandrene* | 0.39+/-0.7 | 0.44+/-0.69 | 1.03+/-0.65 | 4.15+/-3.21 | 1.07+/-1.3 | 0.65+/-0.46 | 2.43+/-3.83 |
| b-pinene* | 0+/-0 | 0.31+/-0.45 | 3.07+/-2.7 | 1.41+/-1.45 | 0.92+/-2.11 | 1.03+/-1.03 | 1.83+/-3.68 |
| limonene* | 1.88+/-2.43 | 0.89+/-0.63 | 5.19+/-6.29 | 8.69+/-3.57 | 1.64+/-2.22 | 2.17+/-1.65 | 4.36+/-6.2 |
| hexanol-2-ethyl | 0.58+/-1.3 | 1.38+/-1.83 | 0.34+/-0.31 | 0+/-0 | 0.72+/-1.01 | 1+/-1.37 | 0+/-0 |
| unknown monoterpene1 | 0.36+/-0.64 | 0.3+/-0.38 | 0.7+/-0.8 | 14.21+/-3.85 | 0.25+/-0.33 | 0.21+/-0.21 | 0.41+/-0.82 |
| menthenol* | 0+/-0 | 0+/-0 | 0.48+/-0.75 | 19.16+/-3.99 | 0+/-0 | 0+/-0 | 0+/-0 |
| p-cymene* | 1.71+/-1.33 | 1.31+/-1.54 | 1.21+/-1.19 | 0.49+/-0.45 | 1.34+/-1.47 | 0.44+/-0.99 | 3.99+/-2.8 |
| camphanol.acetate | 0+/-0 | 0+/-0 | 1.86+/-4.17 | 5.21+/-6.86 | 0+/-0 | 0.48+/-0.8 | 0+/-0 |
| unknown sesquiterpene1 | 0.59+/-1.32 | 1.4+/-2.98 | 1.33+/-2.98 | 0.38+/-0.85 | 0.09+/-0.28 | 0+/-0 | 0+/-0 |
| butoxyethoxy-ethyl-aceate | 0.27+/-0.6 | 1.45+/-2.1 | 0.54+/-1.21 | 0+/-0 | 0.22+/-0.71 | 1.86+/-2.8 | 1.02+/-2.28 |
| a-copaene* | 2.51+/-3.08 | 0.69+/-1.63 | 4.87+/-8.31 | 3.39+/-2.83 | 1.94+/-2.41 | 0.28+/-0.45 | 0.28+/-0.46 |
| boubonene | 0.82+/-1.01 | 0.79+/-2.11 | 8.49+/-17.49 | 2.62+/-2.67 | 0.22+/-0.51 | 2.7+/-2.49 | 0.22+/-0.41 |
| b-copaene* | 1.55+/-1.96 | 2.07+/-4.22 | 1.68+/-3.44 | 2.62+/-4.2 | 0.42+/-0.49 | 0.37+/-0.41 | 0.08+/-0.18 |
| a-bergamotene* | 0.03+/-0.06 | 0+/-0 | 0+/-0 | 0+/-0 | 4.46+/-12.78 | 0+/-0 | 0+/-0 |
| a-caryophyllene* | 0.35+/-0.49 | 0.11+/-0.35 | 4.76+/-10.24 | 2.21+/-2.64 | 0.6+/-1.9 | 2.16+/-1.82 | 0.09+/-0.14 |
| b-cubene | 0.94+/-1.08 | 0.25+/-0.81 | 3.56+/-7.38 | 1.29+/-1.45 | 0.25+/-0.79 | 1.22+/-0.96 | 0.65+/-0.97 |
| unknown hydrocarbon1 | 0.21+/-0.47 | 1.09+/-1.85 | 0+/-0 | 0.29+/-0.65 | 0.88+/-1.2 | 0.83+/-0.81 | 4.59+/-4.68 |
| b-caryophyllene* | 0+/-0 | 0.48+/-1.03 | 2.19+/-2.68 | 4.51+/-5 | 0+/-0 | 0.32+/-0.72 | 0+/-0 |
| a-cubene | 0.08+/-0.17 | 0+/-0 | 8.59+/-19.21 | 4.25+/-5.55 | 0.12+/-0.37 | 0.8+/-1 | 1.01+/-1.64 |
| unknown alkane1 | 0.75+/-1.67 | 1.85+/-3.57 | 0+/-0 | 0+/-0 | 0+/-0 | 0+/-0 | 0+/-0 |
| a-selinene* | 0.69+/-1.55 | 0+/-0 | 2.39+/-5.34 | 4.21+/-2 | 0+/-0 | 0+/-0 | 0+/-0 |
| cadinene* | 0.78+/-1.47 | 0.69+/-1.47 | 2.77+/-4.32 | 1.11+/-1.55 | 0.38+/-0.85 | 0+/-0 | 0+/-0 |
| cubene | 0.11+/-0.24 | 0.58+/-1.07 | 3.54+/-4.11 | 1.09+/-0.56 | 2.41+/-6.86 | 0+/-0 | 0.21+/-0.47 |
| d-cadidene | 0.46+/-0.78 | 0.29+/-0.65 | 2.89+/-6.45 | 9.28+/-3.69 | 0.39+/-0.73 | 0.13+/-0.2 | 0.58+/-0.54 |
| unknown aromatic1 | 0.38+/-0.86 | 1.84+/-2.86 | 0.26+/-0.57 | 0+/-0 | 0.46+/-1.31 | 0+/-0 | 0+/-0 |
| liguloxide | 0+/-0 | 0+/-0 | 0+/-0 | 0+/-0 | 0+/-0 | 7.03+/-5.07 | 0+/-0 |
| tricyclo-decanol | 0.86+/-1.44 | 2.27+/-1.62 | 1.22+/-1.17 | 1.61+/-1.56 | 0.17+/-0.29 | 0.88+/-1.03 | 0.79+/-1.76 |
| cadinol* | 0+/-0 | 0+/-0 | 0.6+/-1.12 | 0.09+/-0.2 | 2.85+/-8.8 | 0+/-0 | 0.86+/-1.62 |
| a-bergamotol* | 0+/-0 | 0+/-0 | 0+/-0 | 0+/-0 | 3.11+/-7.92 | 0+/-0 | 0+/-0 |
| salicylic acid-butyl-ester | 0.28+/-0.21 | 1.19+/-1.55 | 3.51+/-3.92 | 0.08+/-0.17 | 0.32+/-0.3 | 10.3+/-20.21 | 0.95+/-0.83 |
| unknown alkane2 | 0.51+/-1.15 | 1.63+/-1.24 | 1.54+/-1.27 | 0.17+/-0.38 | 1.29+/-0.83 | 1.72+/-1.6 | 0.17+/-0.38 |
| unknown aromatic2 | 2.41+/-1.39 | 3.14+/-1.84 | 4.01+/-1.77 | 2.54+/-1.86 | 2.54+/-1.08 | 2.54+/-0.87 | 1.47+/-0.56 |
| unknown monoterpene2 | 1.69+/-1.51 | 1.81+/-1.07 | 3.87+/-1.01 | 5.57+/-0.99 | 1.63+/-1.14 | 2.42+/-0.43 | 1.94+/-1.31 |
| unknown oxi.monoterpene1 | 0.14+/-0.31 | 0.41+/-0.48 | 2.35+/-1.87 | 3.87+/-1.16 | 0.35+/-0.59 | 0.55+/-0.31 | 0.14+/-0.31 |
| unknow oxy.sesquiterpene1 | 0.92+/-0.65 | 0.37+/-0.48 | 1.11+/-1.2 | 2.58+/-1.51 | 0.55+/-0.89 | 1.29+/-1.05 | 0.74+/-0.77 |
| unknown sesquiterpene2 | 2.83+/-2.56 | 1.5+/-1.63 | 3.56+/-2.7 | 7.44+/-1.91 | 1.7+/-1.44 | 3.15+/-1.26 | 1.94+/-1.23 |

**Table S2.** Probabilities of landing on flowerheads of *Haplopappus* species, including low- (*H. foliosus*) versus high-elevation (*H. scrobiculatus*) for two fly species, *Dioxyna chilensis* and *Trupanea* sp. Shown are the relative probalities of landing calculated out of the total number of landings (e.g., 1 means that all landing happened on the low-elevation flower, while none on the high-elevaiton flower. Zero values mean that no landing on flowers happened during the 10 minutes timeframe of the experiment). Shown are values for both males and females added in pairs (female/male) to the experimental arenas.

| **Fly_species** | **Sex** | **Prop_low** |  | **Fly_species** | **Sex** | **Prop_low** |
| --- | --- | --- | --- | --- | --- | --- |
| *D. chilensis* | female | 1.000 |  | *Trupaena sp.* | female | 1.000 |
| *D. chilensis* | male | 0.714 |  | *Trupaena sp.* | male | 1.000 |
| *D. chilensis* | female | 1.000 |  | *Trupaena sp.* | female | 1.000 |
| *D. chilensis* | male | 1.000 |  | *Trupaena sp.* | male | 0.667 |
| *D. chilensis* | female | 1.000 |  | *Trupaena sp.* | female | 1.000 |
| *D. chilensis* | male | 0.375 |  | *Trupaena sp.* | male | 1.000 |
| *D. chilensis* | female | 1.000 |  | *Trupaena sp.* | female | 0.000 |
| *D. chilensis* | male | 0.750 |  | *Trupaena sp.* | male | 0.000 |
| *D. chilensis* | female | 1.000 |  | *Trupaena sp.* | female | 0.000 |
| *D. chilensis* | male | 0.900 |  | *Trupaena sp.* | male | 1.000 |
| *D. chilensis* | male | 0.000 |  | *Trupaena sp.* | female | 1.000 |
| *D. chilensis* | male | 0.000 |  | *Trupaena sp.* | male | 0.833 |
| *D. chilensis* | female | 1.000 |  | *Trupaena sp.* | female | 1.000 |
| *D. chilensis* | male | 0.750 |  | *Trupaena sp.* | male | 0.000 |
| *D. chilensis* | female | 1.000 |  | *Trupaena sp.* | female | 0.000 |
| *D. chilensis* | male | 1.000 |  | *Trupaena sp.* | male | 0.000 |
| *D. chilensis* | female | 1.000 |  | *Trupaena sp.* | female | 0.000 |
| *D. chilensis* | male | 1.000 |  | *Trupaena sp.* | male | 0.000 |
| *D. chilensis* | female | 1.000 |  | *Trupaena sp.* | female | 0.000 |
| *D. chilensis* | male | 1.000 |  | *Trupaena sp.* | male | 0.000 |
| *D. chilensis* | female | 0.500 |  |  |  |  |
| *D. chilensis* | male | 0.000 |  |  |  |  |
| *D. chilensis* | female | 1.000 |  |  |  |  |
| *D. chilensis* | male | 1.000 |  |  |  |  |
| *D. chilensis* | female | 0.750 |  |  |  |  |
| *D. chilensis* | male | 0.538 |  |  |  |  |
| *D. chilensis* | female | 1.000 |  |  |  |  |
| *D. chilensis* | male | 1.000 |  |  |  |  |
| *D. chilensis* | female | 1.000 |  |  |  |  |
| *D. chilensis* | male | 0.000 |  |  |  |  |

**Figures**

**Figure S1**. Distribution maps of seven *Haplopappus* species surveyed in the study. Pictures on the right show, from top to bottom, *H. anthylloides*, *H. uncinatus*, and *H. foliosus*. Distribution ranges were obtained from Klingenberg {, 2007 #3991}. Map on the left shows the main geographic area of Central Chile, where the study was conducted, and with Santiago (red dot) as reference point.


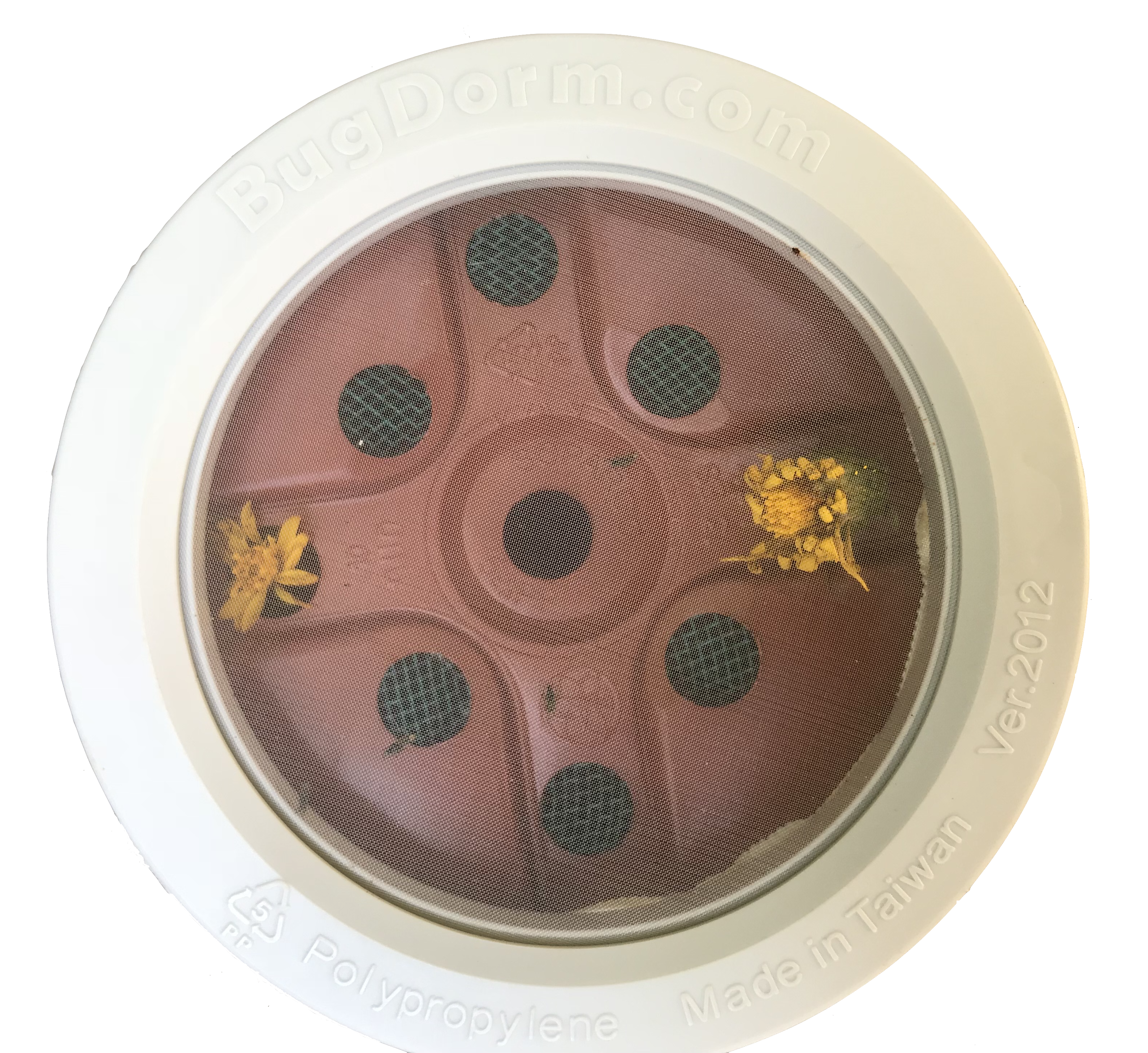


**Figure S2.** Insect preference bioassay design. We constructed seven arenas by taping a round BugDorm mesh lid to the bottom of an inverted a plastic pot. *Dioxyna* and *Trupanea* flies were collected from all elevations and tested in male-female pairs in an arena with *H. foliosus* (right) from low elevation and *H. scrobiculatus* (left) from high elevation. One male and one female fly were paired and given ten minutes as number of landings was counted for each high or low elevation treatment.

**
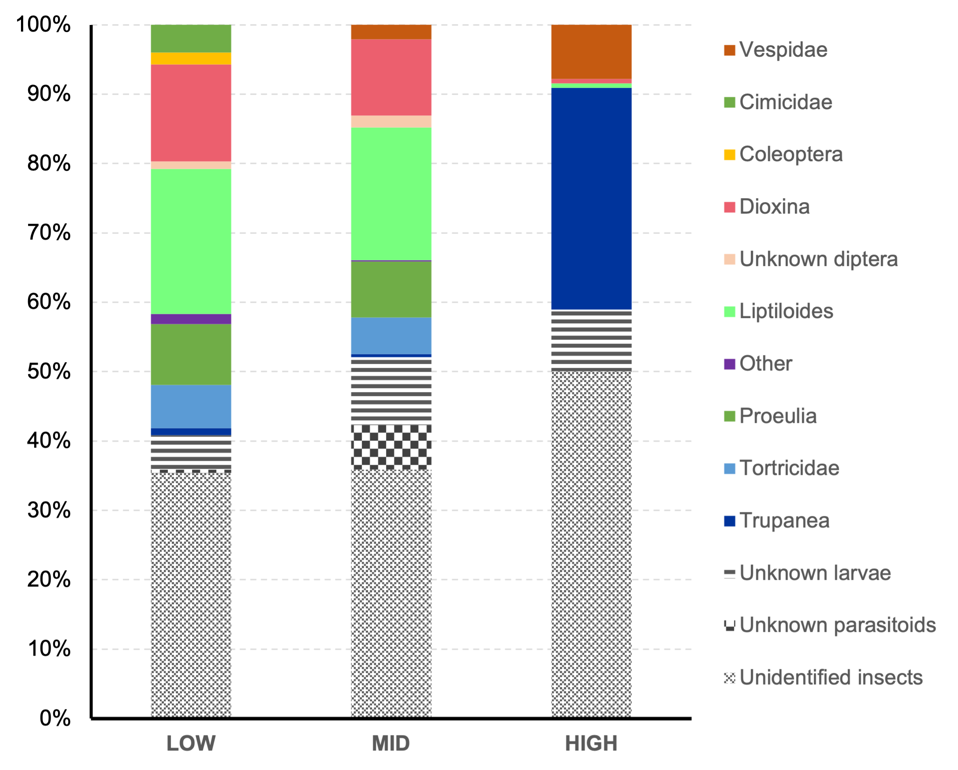
**

Relative insect abundance

**
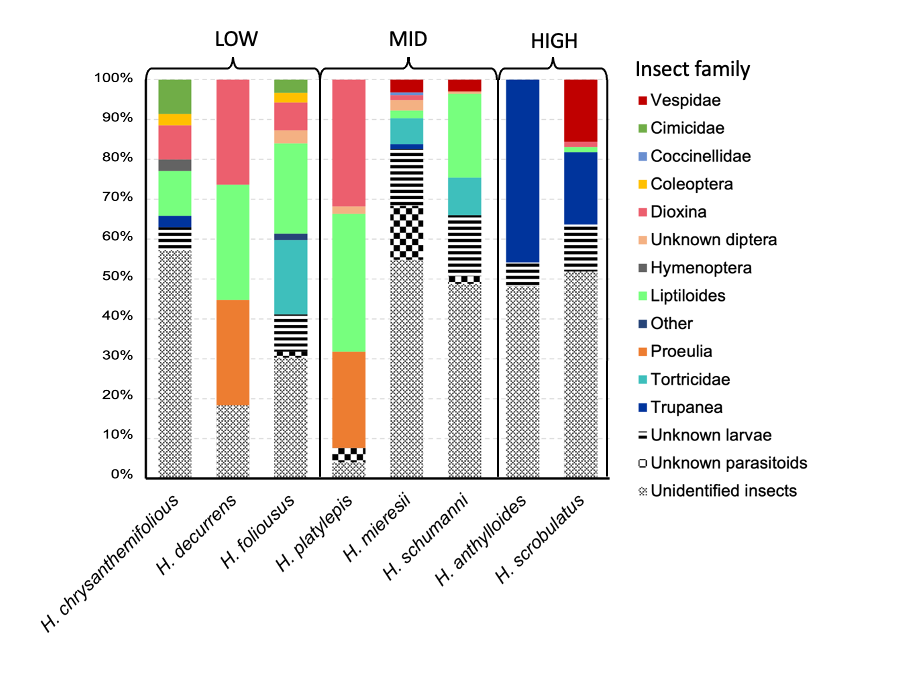
**

Relative insect abundance

**Figure S3**. Insect diversity found in the *Haploppapus* system species by elevational bands (top panel) and by *Haplopappus* species (low panel). Insects developing within the inflorescent consisted of seed and insect parasitoids. Most common insect species were identified at the genus level and the rest to the family level. Identified insects are colour coded while unknown or unidentified insects are indicated by greyscale, fill pattern.
